# *Helleborus purpurascens*—Amino Acid and Peptide Analysis Linked to the Chemical and Antiproliferative Properties of the Extracted Compounds

**DOI:** 10.3390/molecules201219819

**Published:** 2015-12-11

**Authors:** Adina-Elena Segneanu, Ioan Grozescu, Florentina Cziple, Daniel Berki, Daniel Damian, Cristina Mariana Niculite, Alexandru Florea, Mircea Leabu

**Affiliations:** 1 National Institute for Research and Development in Electrochemistry and Condensed Matter—INCEMC, Timisoara 300224, Romania; ioangrozescu@gmail.com (I.G.); danieldamian83@gmail.com (D.D.); 2 University Politehnica Timisoara, 2 Piata Victoriei, Timisoara 300006, Romania; berki_daniel@yahoo.com; 3 University “Eftimie Murgu”, Resita, 1-4 Traian Vuia, Resita 320085, Romania; cflorentina@yahoo.com; 4 Department of Morphological Sciences, University of Medicine and Pharmacy “*Carol Davila*”, 8 Eroilor Sanitari Blvd, Sector 5, Bucharest 050474, Romania; maria.niculite@ivb.ro (C.M.N.); alexflorea1992@gmail.com (A.F.); mircea.leabu@ivb.ro (M.L.); 5 “Victor Babes” National Institute of Pathology, 99-101, Splaiul Independentei, Sector 5, Bucharest 050096, Romania

**Keywords:** antiproliferative activiy, chromatographic techniques, hellebore, thionins, mass spectroscopy

## Abstract

There is a strong drive worldwide to discover and exploit the therapeutic potential of a large variety of plants. In this work, an alcoholic extract of *Helleborus purpurascens* (family *Ranunculaceae*) was investigated for the identification of amino acids and peptides with putative antiproliferative effects. In our work, a separation strategy was developed using solvents of different polarity in order to obtain active compounds. Biochemical components were characterized through spectroscopic (mass spectroscopy) and chromatographic techniques (RP-HPLC and GC-MS). The biological activity of the obtained fractions was investigated in terms of their antiproliferative effects on HeLa cells. Through this study, we report an efficient separation of bioactive compounds (amino acids and peptides) from a plant extract dependent on solvent polarity, affording fractions with unaffected antiproliferative activities. Moreover, the two biologically tested fractions exerted a major antiproliferative effect, thereby suggesting potential anticancer therapeutic activity.

## 1. Introduction

Natural products isolated/extracted from medicinal plants have high potential as new bioactive compounds. As a result, molecules with both low molecular weight (e.g., quinones, cerebrosides, isoflavones, catechol, amines, terpenes, steroids, *etc.*) and high molecular weight (e.g., polysaccharides, glycoproteins, glycopeptides, proteins, *etc.*) have been investigated as putative pharmaceutical agents [1].

*Helleboreus* spp. (family *Ranunculaceae*) are spontaneous perennial flowering herb plants. In the Romanian spontaneous flora, there are two species of hellebore: *Helleborus purpurascens* and *Helleborus odorus*, both of them flowering in early spring [2,3]. *Helleborus purpurascens* is extremely toxic, yet known since long ago for its folkloric therapeutic effects. It has been used in traditional medicine since ancient times for the treatment of various diseases: pain, mental and cardiac disorders, *etc.* References on the therapeutic use of this herb go back to the time of Hippocrates. In the Middle Ages, the physician and alchemist Paracelsus prepared and proposed an extract of *Helleborus* as an “elixir” to increase life expectancy [4]. The Romanian researcher Boici used a purified hellebore extract for the preparation of a pharmaceutical product named Boicil, which showed therapeutic activity in rheumatic diseases [5]. The plant extract is used as an anti-rheumatic ointment without any previous investigation of the activity of the individual components of hellebore, therefore, the component(s) responsible for the biological activity of Boicil have not been identified yet. However, recent studies on the antitumor, immune and biological activity of *Helleborus* extracts revealed the multitude of directions in this field of research targeting the elucidation of the biological potential and cellular mechanisms addressed by various components from this plant [6,7,8]. Some of the main components of *Helleborus purpurascens* are glycosides, thionins (helleborine, hellebrine), saponides, resins, lactones, protoanemonine, and minerals [6,7,8,9,10].

The significance of studying the structure of natural peptides was illustrated by numerous pharmacological investigations, identifying various essential properties of these types of compounds such as their low molecular mass, their relatively unique and simple structures, low antigenicity, low toxicity, and an highly biological activity because of their remarkable chemical diversity, steric complexity and high biochemical specificity [1,11,12,13]. Peptide properties depend on the constituent amino acids and the amino acid sequences in the peptide structure. Modern chemistry offers various methods for biochemical and structural analysis of peptides and glycopeptides. Chromatography and mass spectrometry proved to be efficient techniques for the identification of each component from a complex natural matrix [14].

Thionins are relatively small-sized multiple-cysteine peptides with antimicrobial properties due to their high content of cysteine units [15]. The antitumor and immunomodulating activities of hellebore are attributed to the cytotoxicity of this natural peptide [15,16,17,18,19]. Although there are many studies worldwide on hellebore extracts and their biological activities, research in this area continues to be of great interest because the chemical structure and composition of *Helleborus purpurascens* have not been fully elucidated yet [6,20,21,22,23]. The aim of this study was hence to isolate the amino acids and thionins from an alcoholic extract of *Helleborus purpurascens*, identify them using spectroscopic and chromatographic techniques and to test their biological activity in terms of antiproliferative effects.

## 2. Results and Discussion

A mandatory step toward separation of target compounds in an extract is the development of an appropriate partition scheme sequence for phytochemicals, employing different types of solvents, in order to remove metabolites and unwanted compounds, such as: lipids, phenols, detergents, resins, lactones, protoanemonins. Depending on the polarity of the solvent used, there emerges a wide distribution of phytochemicals from the hellebore extract in the different fractions recovered [24,25,26,27].

### 2.1. HPLC Analysis

The presence of amino acids in hellebore extracts was examined using HPLC. The influence of the solvent (polarity) on the amino acid content in the analyzed samples (S_1_, S_4_, S_5_ and S_6_) was also taken under consideration. Hence, a qualitative reverse phase HPLC analysis (RP-HPLC) for free amino acids was investigated. Under the same chromatographic conditions, a phenylisothiocyanate (PITC) pre-column derivatization method for standard amino acids was developed. The derivatization step, as a widely used procedure for HPLC analysis of amino acids and peptides, is not necessary if chromatography aims only at separating the amino acids. Comparison of the results of the pre-column PITC derivatization methods for HPLC analysis with those of the RP-HPLC analysis of the hellebore extract could identify its amino acid composition. The proposed RP-HPLC method enabled identification of the amino acids existing in the hellebore fractions S_1_, S_4_, S_5_ and S_6_ (see Table 1).

The developed RP-HPLC method allowing for separation and identification of the amino acids in the hellebore extract (S_1_, S_4_, S_5_ and S_6_) is based on the retention times of the used standards.

**Table 1:** RP-HPLC identification of *Helleborus purpurascens* amino acids.

Hellebore Fraction	Amino Acids	Retention Time (Tr) (min)	Peak Area (mAu·min)	Peak High (mAu)	Relative Area
S_1_ (ethanolic)	Cys	6.319	19.7050	48.247	12.79
	His	7.21	0.6849	13.520	5.00
	Phe	7.978	9.4757	15.515	6.15
	Thr	8.923	0.9001	3.720	5.95
	Glu	9.958	6.4976	10.478	4.22
	Asn	22.301	108.2632	130.637	88.89
	Ala	32.290	117.4534	110.475	76.25
S_4_ (CH_2_Cl_2_)	Gly	6.987	0.7801	3.567	2.39
	His	7.21	0.85	14.359	4.23
	Phe	7.89	7.854	21.464	6.43
	Glu	9.958	6.891	11.872	5.27
	Ser	12.892	1.5890	9.671	13.561
	Asn	22.301	9.376	5.701	8.429
	Pro	29.862	1.8539	2.257	23.13
	Ala	32.290	0.9369	1.457	9.22
	Lys	35.802	3.2473	3.401	19.28
	Tyr	39.347	0.5518	0.743	8.37
S_5_ (*n*-butanol)	Gly	6.987	8.627	8.672	10.391
	His	7.21	0.9138	12.188	14.57
	Phe	7.978	5.624	9.297	4.61
	Glu	9.958	0.5390	0.948	7.94
	Arg	13.680	0.5313	0.860	17.19
	Val	25.068	0.7673	0.754	14.83
	Ala	32.523	0.3391	0.382	9.97
	Lys	35.802	3.287	11.720	8.134
	Tyr	39.347	10.346	23.581	12.365
S_6_ (aqueous)	C-C	6.198	12.0542	59.209	6.52
	Phe	7.978	27.659	56.375	29.991
	Asp	23.546	15.7821	121.368	8.53
	Ala	32.523	157.0749	139.018	54.95

### 2.2. GC-MS Analysis

**Figure 1:**
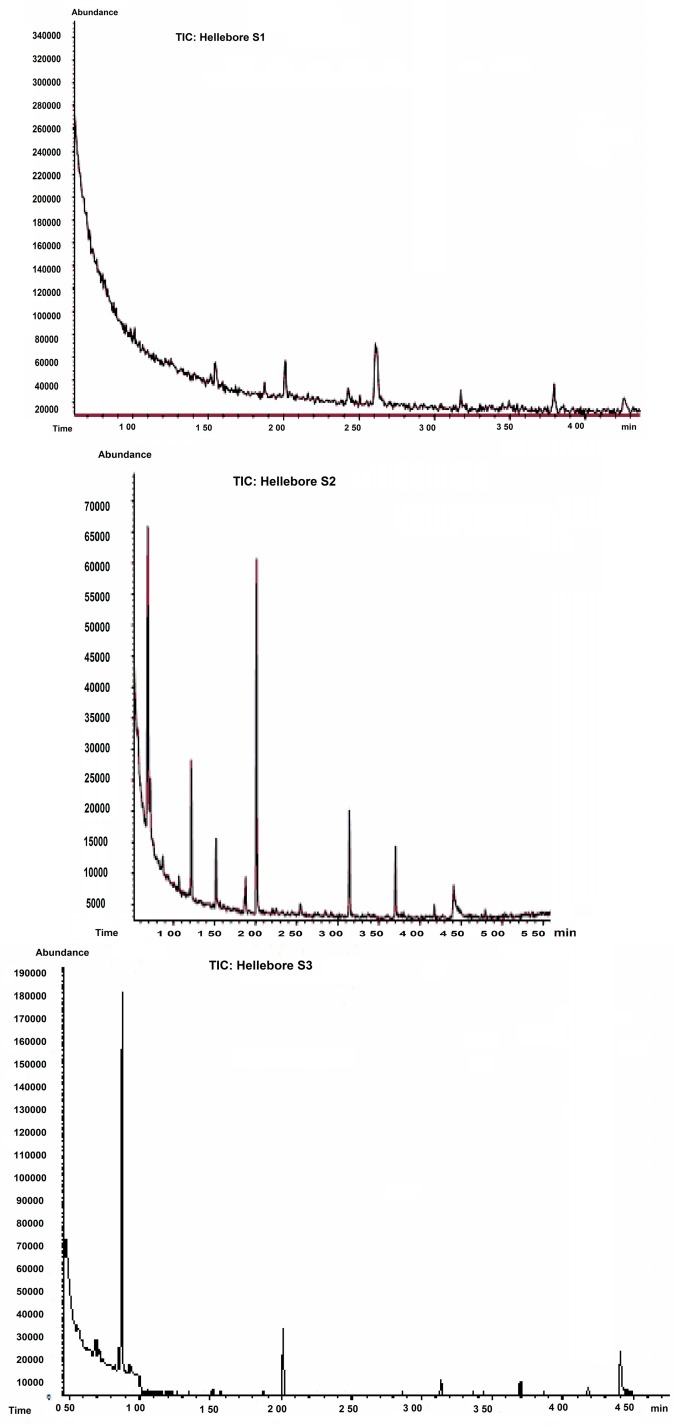

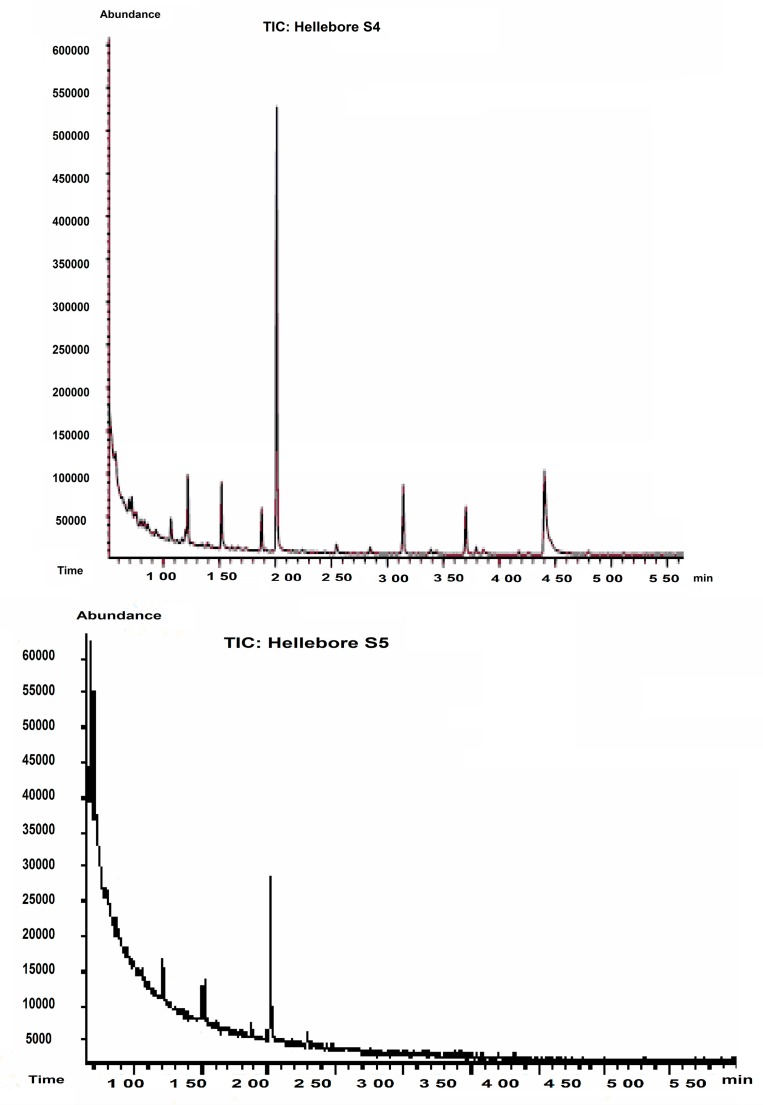
GC-MS chromatogram for S_1_–S_5_ hellebore extract.

The content of hellebore extracts in amino acids and peptides and its dependence on solvent type were confirmed by GC-MS analysis. The obtained chromatograms are shown in Figure 1. The mass spectra of the components from the GC-MS chromatograms were compared with those from the NIST/NBS (National Institute of Standards and Technology/National Bureau of Standards**)** spectral database, and the identified amino acids and peptides are presented in Table 2.

The proposed isolation strategy unraveled the influence of solvent polarity on the efficiency of amino acid and peptide separation from the hellebore extract. Thus, the hexane fraction (S_2_) contains the largest number of amino acids, with the fewest compounds separated in the ethanol fraction (S_1_).

**Table 2:** GC-MS amino acid identification.

Hellebore Fraction	Proposed Structure	Abbreviation	SIM (Selected-Ion Monitoring)
S_1_ (ethanol)	cystine	C-C	41, 42
	glutamic acid	Glu	38, 40
	phenylalanine	Phe	56, 57
	hystidine	Hys	84
	asparagine	Asn	155, 69
	isoleucine	Ile	170, 130
	threonine	Thr	180
	β-alanine	β-Ala	216, 129
S_2_ (hexane)	glutamic acid	Glu	38, 40
	cystine	C-C	41, 42
	ornitine	Orn	59, 60, 61
	isoleucine	Ile	170, 130
	hystidine	Hys	84
	proline	Pro	211
	valine	Val	158, 72
	β-alanine	β-Ala	216, 129
	threonine	Thr	180
	homoserine	HSER	102, 128, 143
	asparagine	Asn	155, 69
	α-aminopimelic acid	APA	198, 258, 286
	arginino-succinic acid	ARG-SUC	326
S_3_ (CCl_4_)	asparagine	Asn	155, 69
	alanine	Ala	130, 70
	glutamic acid	Glu	38, 40
	phenylalanine	Phe	59, 60
	homoserine	HSER	102, 128, 143
	threonine	Thr	180
	lysine	Lys	170, 129
	glicine	Gly	116, 74
	glycyl-glycine (dipeptide)	Gly-Gly	117, 144, 201
	proline-hydroxyproline (dipeptide)	PHP	156, 186
	lysine-alanine (dipeptide)	Lys-Ala	170, 224, 153
	hystidine	Hys	84
	2,4-diamino-*n*-butyric acid	DABA	203, 142, 245
	1-methylhistidine	1MHIS	298
	arginino-succinic acid	ARG-SUC	326
	α-aminopimelic acid	APA	198, 258, 286
S_4_ (CH_2_Cl_2_)	valine	Val	158, 72
	β-alanine	β-Ala	216, 129
	isoleucine	Ile	170, 130
	glutamic acid	Glu	38, 40
	phenylalanine	Phe	59, 60
	serine	Ser	146,9
	tyrosine	Tyr	61, 63
	proline-hydroxyproline (dipeptide)	PHP	156, 186
	lysine	Lys	170, 129
	glycine	Gly	116, 74
	glutamine	Gln	83, 186
	hystidine	His	84
	asparagine	Asn	155, 69
S_5_ (*n*-butanol)	glutamic acid	Glu	38, 40
	phenylalanine	Phe	59, 60, 76
	tyrosine	Tyr	61, 63, 94
	hystidine	His	84, 115
	glycine	Gly	116, 74
	lysine	Lys	170, 129
	valine	Val	158, 72

### 2.3. Hellebore Thionins Characterized by GC-MS

**Figure 2:**
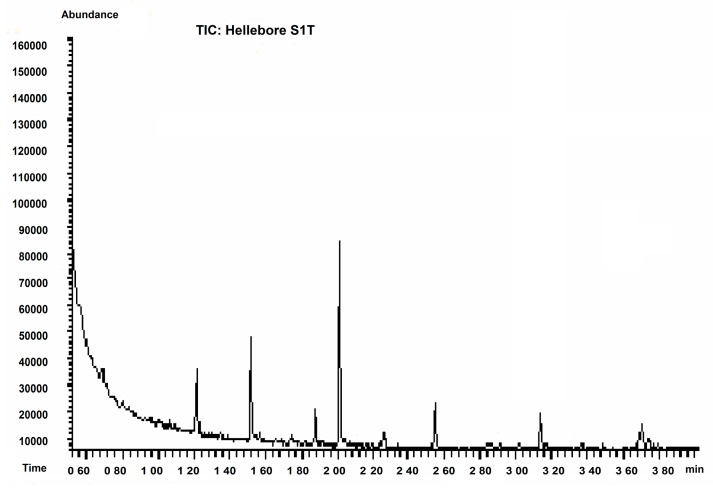

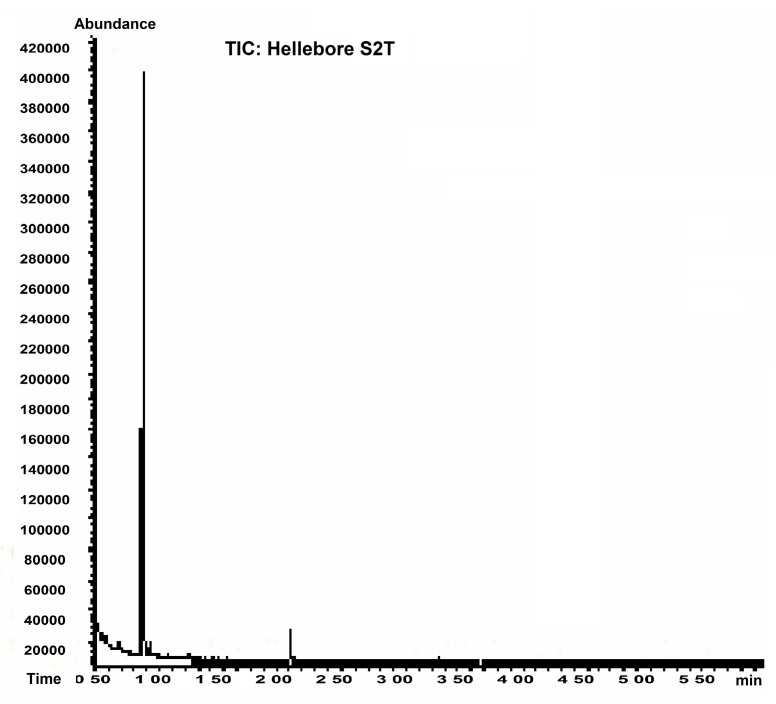
GC-MS chromatogram for S_1T_ and S_2T_ hellebore extract.

The results of the GC-MS for samples S_1T_ (petroleum ether) and S_2T_ (acetone) are shown in Figure 2.

The compounds identified by mass spectral interpretation through GC-MS analysis are listed in Table 3.

The efficiency of the chosen separation strategy for thionins was evaluated by GC-MS analysis. The results show the influence of solvent polarity on the number of isolated compounds. In the low polarity fraction S_1T_ (petroleum ether); eleven amino acids and one dipeptide were isolated and identified in the GC-MS chromatogram (Table 3).

**Table 3:** Thionin GC-MS identification.

Hellebore Fraction	Proposed Structure	Abbreviation	SIM
S_1T_ (petroleum ether)	cystine	C-C	41, 42
	histidine	His	84, 115
	tyrosine	Tyr	61, 63, 94
	lysine	Lys	170, 129
	homoserine	HSER	102, 128, 143
	β-alanine	β-Ala	216, 129
	leucine	Leu	172, 86
	phenylalanine	Phe	59, 60, 76
	glutamic acid	Glu	38, 40
	glycine	Gly	116, 74
	3-methylcysteine	-	172, 259, 130
	proline-hydroxyproline (dipeptide)	PHP	156, 186
S_2T_ (acetone)	cystine	C-C	41, 42
	glutamic acid	Glu	38, 40
	tyrosine	Tyr	61, 63, 94
	valine	Val	158, 72
	β-alanine	β-Ala	216, 129
	phenylalanine	Phe	59, 60, 76

### 2.4. Nano-ESI-Chip-MS Analysis

**Figure 3:**
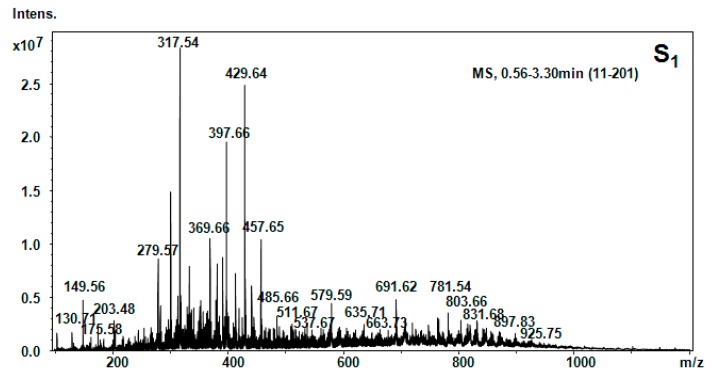
Positive ion mode nano-ESI-Chip-MS forS_1_ hellebore fraction.

**Figure 4:**
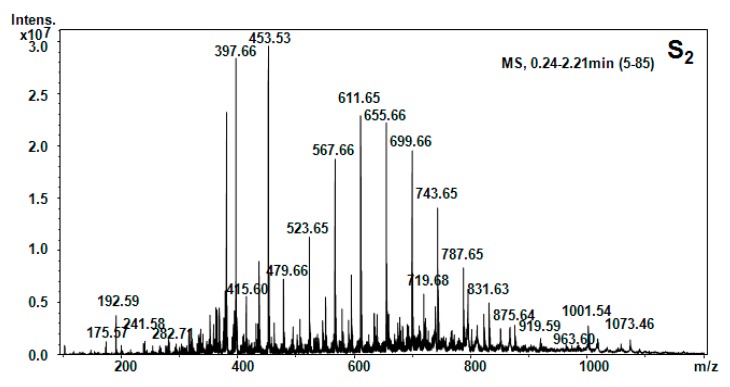
Positive ion mode nano-ESI-Chip-MS for S_2_ hellebore fraction.

**Figure 5:**
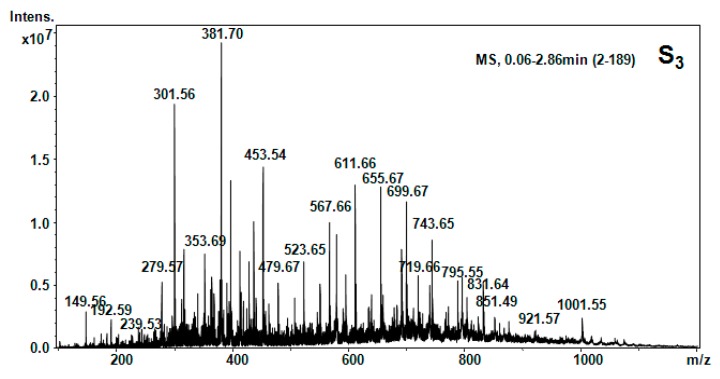
Positive ion mode nano-ESI-Chip-MS for S_3_ hellebore fraction.

**Figure 6:**
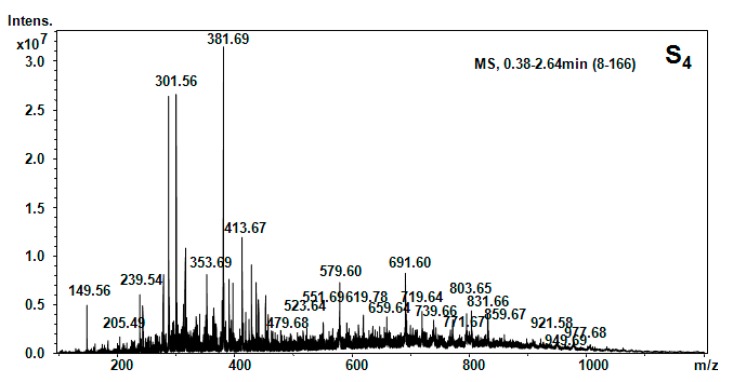
Positive ion mode nano-ESI-Chip-MS for S_4_ hellebore fraction.

**Figure 7:**
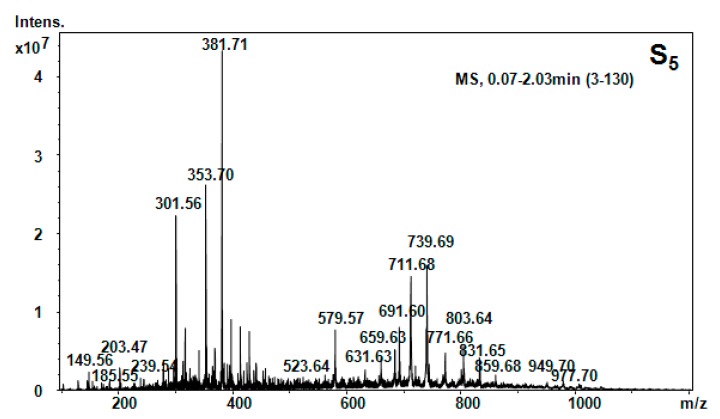
Positive ion mode nano-ESI-Chip-MS for S_5_ hellebore fraction.

**Figure 8:**
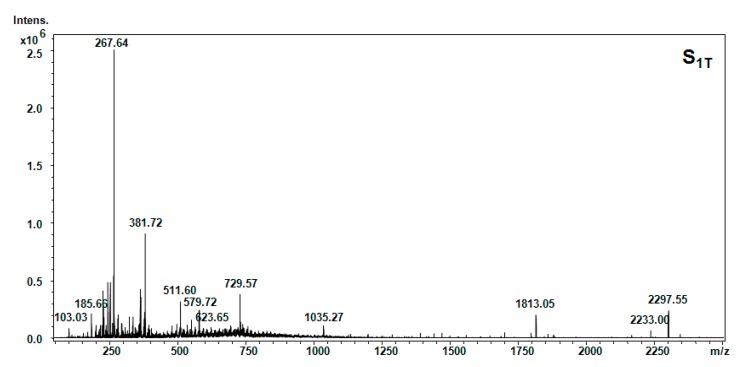
Positive ion mode nano-ESI-Chip-MS of the hellebore thionins in the petroleum ether fraction.

**Figure 9:**
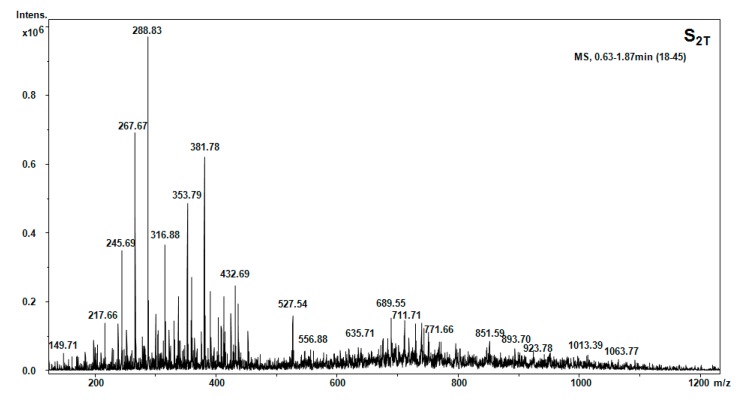
Positive ion mode nano-ESI-Chip-MS of the hellebore thionins in acetone fraction.

All five hellebore fractions S_1_–S_5_ were submitted to high-throughput positive nanoESI chip MS and screening under identical solution and instrumental parameters. The obtained mass spectra are illustrated in Figure 3, Figure 4, Figure 5, Figure 6, Figure 7, Figure 8 and Figure 9, while the ion fragments and the corresponding amino acids and peptides (assignments to amino acids and peptide structures) are listed in Table 4.

**Table 4:** Amino acids and peptides species in hellebore fractions detected by MS.

Hellebore Fraction	Type of Molecular Ion	*m*/*z* Detected	*m*/*z* Calculated	Proposed Structure
S_1_ (ethanol)	[M + 2H]^2+^	149.56	147.18	Phe
	[M + 2H]^2+^	130.71	128.14	Gln
	[M + 2H]^2+^	175.58	173.4	Arg
	[M + NH_3_]^+^	203.48	203.14	Tryptophan
	[M + H_2_O + 2H]^2+^	279.57	279.34	Cys-Arg
	[M + 2H]^2+^	317.54	317.3	Asp-Glu-β-Ala
	[M + 2H]^2+^	369.66	369.37	His-Glu-Thr
	[M + H_2_O + 2H]^2+^	397.66	397.44	C-C-His
	[M + H]^+^	429.64	429.46	Asn-β-Ala-Ile-Glu
	[M + H]^+^	457.65	457.52	Phe-His-Thr-β-Ala
	[M + H_2_O]	579.59	579.67	Thr-Phe-Ile-Glu-β-Ala
	[M + H_2_O + H^+^	691.62	691.8	Arg-C-C-Phe-Glu
S_2_ (hexane)	[M + 2H]^2+^	175.58	173.4	Arg
	[M + H_2_O + 2H]^2+^	192.59	192.39	β-Ala-Thr
	[M + H_2_O + 2H]^2+^	397.66	397.44	C-C-His
	[M + H_2_O + H]^+^	415.6	415.48	Glu-Pro-Val-β-Ala
	[M + H_2_O + H]^+^	453.53	453.5	Ser-His-Ile-Pro
	[M + H_2_O + H]^+^	479.66	479.54	Apa-Ile-Thr-β-Ala
	[M + H_2_O + 2H]^2+^	523.65	523.5	Hser-Orn-His-Asn
	[M + NH_3_]^+^	611.65	611.72	Arg-C-C-Thr-Pro
	[M + 2H]^2+^	655.66	655.6	Arg-Suc-Asn-HSer-Glu
	[M + H_2_O + H]^+^	743.65	743.68	Arg-Succ-Asn-HSer-Glu-β-Ala
	[M + H_2_O + H]^+^	729.57	729.39	Gly-Cystine-Met-Tyr-Hser
S_3_ (CCl_4_)	[M + 2H]^2+^	149.56	147.18	Phe
	[M + H_2_O + 2H]^2+^	192.59	192.39	β-Ala-Thr
	[M + H_2_O + 2H]^2+^	279.57	279.34	Cys-Arg
	[M + H]^+^	301.56	301.56	1MHis-Gly-Gly
	[M + NH_3_]^+^	381.70	381.32	Lys-Ala-Phe
	[M + H]^+^	453.54	453.44	His-Glu-Asn-β-Ala
	[M + H_2_O + H]^+^	479.66	479.54	APA-Ile-Thr-β-Ala
	[M + H_2_O + 2H]^2+^	523.65	523.5	HSer-Orn-His-Asn
	[M + H_2_O + H]^+^	552.98	552.62	Pro-Hyp-Phe-Thr-Gly
	[M + NH_3_]^+^	611.65	611.72	Arg-C-C-Thr-Pro
	[M + 2H]^2+^	655.67	655.6	Arg-Suc-Asn-HSer-Glu
	[M + H_2_O + H]^+^	743.65	743.68	Arg-Suc-Asn-HSer-Glu-β-Ala
	[M + H_2_O + H]^+^	729.57	729.39	Gly-Cystine-Met-Tyr-Hser
S_4_ (CH_2_Cl_2_)	[M + 2H]^2+^	149.56	147.18	Phe
	[M + H_2_O + H]^+^	205.49	205.42	Val-Ser
	[M + H_2_O + H]^+^	239.54	239.27	Gly-Tyr
	[M + H_2_O + 2H]^2+^	279.57	279.34	Cys-Arg
	[M + H]^+^	301.56	301.56	1MHis-Gly-Gly
	[M + H_2_O + 2H]^2+^	353.69	353.39	Gly-Tyr-Ile
	[M + NH_3_]^+^	381.69	381.32	Lys-Ala-Phe
	[M + NH_3_]^+^	413.67	413.46	Hyp-Lys-His
	[M + H_2_O + H]^+^	415.6	415.48	Glu-Pro-Val-β-Ala
	[M + H_2_O + H]^+^	479.66	479.54	Apa-Ile-Thr-β-Ala
	[M + H_2_O + H]^+^	552.98	552.62	Pro-Hyp-Phe-Thr-Gly
	[M + H_2_O + H]^+^	579.59	579.67	Thr-Phe-Ile-Lys-β-Ala
	[M + H_2_O + 2H]^2+^	619.78	619.80	His-Ser-Val-Tyr-Ile
	[M + H_2_O + H]^+^	691.62	691.8	Arg-C-C-Phe-Glu
	[M + H_2_O + H]^+^	719.64	719.80	His -Ser-Val-Tyr-Ile-Thr
S_5_ (*n*-butanol)	[M + 2H]^2+^	149.56	147.18	Phe
	[M + NH_3_]^+^	203.48	203.14	Trn
	[M + H]^+^	301.56	301.56	1MHis-Gly-Gly
	[M + H_2_O + 2H]^2+^	353.69	353.39	Gly-Tyr-Ile
	[M + NH_3_]^+^	381.69	381.32	Lys-Ala-Phe
	[M + H_2_O + 2H]^2+^	523.65	523.5	HSer-Orn-His-Asn
	[M + H_2_O + H]^+^	579.59	579.67	Thr-Phe-Ile-Lys-β-Ala
	[M + H_2_O + 2H]^2+^	619.78	619.80	His-Ser-Val-Tyr-Ile
	[M + H_2_O + H]^+^	691.62	691.8	Arg-C-C-Phe-Glu
S_1T_ (petroleum ether)	[M + 2H]^2+^	103.03	103.11	Thr
	[M + H]^+^	104.3	104.15	Cys
	[M + H]^+^	185.66	185.25	β-Ala-Ile
	[M + NH_3_]^+^	203.48	203.14	Trn
	[M + NH_3_]^+^	267.64	267.34	Phe-Cys
	[M + NH_3_]^+^	381.69	381.32	Lys-Ala-Phe
	[M + H_2_O + 2H]^2+^	511.60	511.35	3-methyl-Cys-Hyp-Pro-Gln
	[M + H_2_O + H]^+^	552.98	552.62	Pro-Hyp-Phe-Thr-Gly
	[M + H_2_O + H]^+^	579.59	579.67	Thr-Phe-Ile-Glu-β-Ala
	[M + H_2_O + H]^+^	691.62	691.8	Arg-C-C-Phe-Glu
	[M + H_2_O + H]^+^	729.57	729.39	Gly-C-C-Met-Tyr-HSer
S_2T_ (acetone)	[M + 2H]^2+^	103.03	103.11	Thr
	[M + H]^+^	104.3	104.15	Cys
	[M + H]^+^	185.66	185.25	β-Ala-Ile
	[M + NH_3_]^+^	203.48	203.14	Trn
	[M + NH_3_]^+^	267.64	267.34	Phe-Cys
	[M + H_2_O + 2H]^2+^	288.83	288.33	Met-His
	[M + H]^+^	301.56	301.56	1MHis-Gly-Gly
	[M + H_2_O + 2H]^2+^	353.69	353.39	Gly-Tyr-Ile
	[M + NH_3_]^+^	381.69	381.32	Lys-Ala-Phe
	[M + H_2_O + 2H]^2+^	432.69	432.46	Phe-His-Gly-β-Ala
	[M + H_2_O + 2H]^2+^	523.65	523.5	HSer-Orn-His-Asn
	[M + H_2_O + 2H]^2+^	527.54	527.60	Orn-Val-Glu-Phe
	[M + H_2_O + H]^+^	552.98	552.62	Pro-Hyp-Phe-Thr-Gly
	[M + H_2_O + H]^+^	579.59	579.67	Thr-Phe-Ile-Glu-β-Ala
	[M + H_2_O + H]^+^	689.55	689.35	Ile-C-C-Trp-Hyp
	[M + H_2_O + H]^+^	691.62	691.8	Arg-C-C-Phe-Glu
	[M + H_2_O + H]^+^	729.57	729.39	Gly-C-C-Met-Tyr-HSer

To elucidate the chemical composition of the hellebore fractions, we have developed a fully automated new mass spectrometry method based on nanoESI high-capacity ion trap (HCT), in a similar manner as described in the literature [28]. The mass spectrometry results confirmed the chemical structures detected by chromatographic techniques. Although, the developed chromatographic analysis method offered information about the great majority of the chemical species present in the hellebore extracts, mass spectrometry provided many more data on the complexity of the protein structures found in the studied hellebore fractions. Comparison of the screened mass spectra of the hellebore fractions S_1_–S_5_ provided direct evidence of the variable composition of each fraction due, primarily, to the solvent employed. The investigation of thionin composition from the hellebore fractions S_1T_ and S_2T_ highlighted the same compounds identified by previous analytical methods. The information gained from this study corroborates with the existing data from the literature [29,30]. Our results revealed that the proposed methods proved to be useful tools for the separation and identification of individual compounds from complex natural mixtures.

### 2.5. Biological Activity Investigation

**Figure 10:**
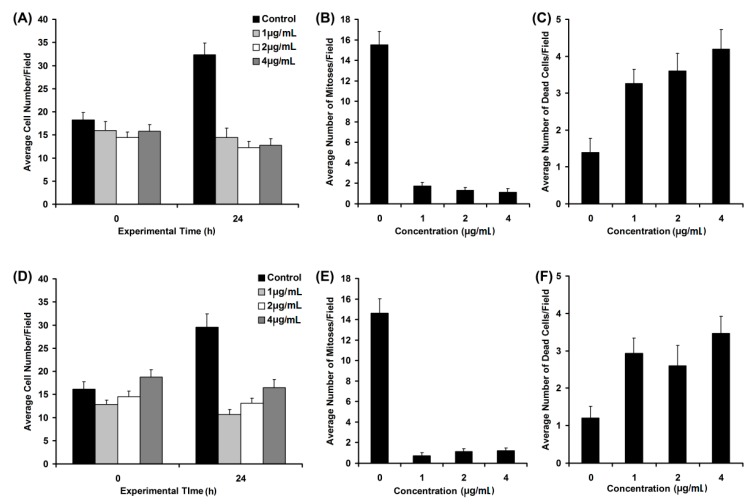
Effects of S_5_ (**A**–**C**) and S_2T_ (**D**–**F**) fractions on HeLa cell behavior monitored by timelapse videomicroscopy. Untreated cells have grown as usual, doubling their number in 24 h (**A**–**D**, control black bars).

The usefulness of timelapse videomicroscopy in the investigation of cell behavior and biological activity of different pharmaceuticals was previously argued upon and proven [31,32]. Here, studies were conducted to assess, in a cancer cell line (HeLa), the effects of two fractions obtained from the alcoholic extract of *Helleborus purpurascens*. As shown in Figure 10, the cells treated with various concentrations of S_5_ (Figure 10A) and S_2T_ (Figure 10D) failed to multiply. The number of mitoses significantly decreased for treated cells for all concentrations used of both S_5_ (Figure 10B) and S_2T_ (Figure 10E). The number of dead cells did not spectacularly increase, although it varied significantly in comparison to untreated cells for both S_5_ (Figure 10C) and S_2T_ (Figure 10F).

**Figure 11:**
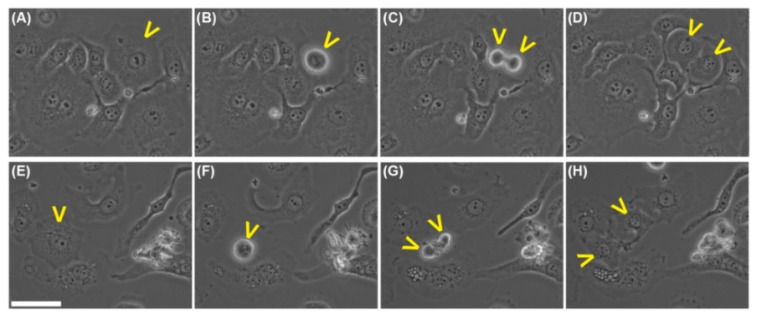
Phase contrast micrographs showing cells before, during and at the end of mitosis. Yellow arrowheads indicate cells of interest. (**A**) Control cells before starting any mitosis; (**B**) A control cell is rounding up starting its mitosis; (**C**) The same cell as in (**B**) 20 min later, during cytokinesis; (**D**) The two daughter cells, resulting in mitosis, significantly spread 1 h after mitosis was started; (**E**) Treated cells before starting any mitosis; (**F**) A treated cell rounded up and starting its mitosis; (**G**) The same cell as in (**F**), 200 min later, accomplishing its cytokinesis; (**H**) The daughter cells showing significant spreading 90 min later, that means almost 5 h after mitosis has started. White scale bar in (**E**) represents 50 μm, and is identical for all the images.

Both tested fractions (S_5_ and S_2T_) inhibited cell proliferation at significantly low concentrations, as shown by HeLa cell growth in the absence or presence of compounds (Figure 10A,D). Cells that were not treated proliferated and doubled their number after a 24 h monitoring. The number of observed mitoses was significantly higher in the absence of the tested compounds (Figure 10B,E). It is noteworthy that the number of dead cells during the experimental period of 24 h, although significantly increased after treatment, still remained very low (Figure 10C,F), indicating a reduced cytotoxic effect of both fractions S_5_ and S_2T_, isolated from the *Helleborus purpurascens* alcoholic extract. With regard to the dynamics of the identified effects, fraction S_5_ arrested cell division almost completely after 6 h, when the highest concentration was used. For the medium and low concentration, the same effect was observed at about 12 h and beyond. A very interesting result was observed in terms of kinetics of the few mitoses still occurring in the treated samples. If for untreated HeLa cells a starting mitosis succeeded finalizing its cytokinesis stage in no more than half an hour, treated cells starting mitosis needed a significantly longer period until cytokinesis occurred, meaning more than 3 h (Figure 11).

Accordingly, an untreated cell, spread on the culture surface (Figure 11A), started a mitotic process (Figure 11B) and accomplished cytokinesis after 20 min (Figure 11C), whereas the two daughter cells had already spread, 1 h after mitosis has begun (Figure 11D). A treated cell (Figure 11E) that entered mitosis (Figure 11F) needed 200 min to accomplish cytokinesis (Figure 11G), and 90 min more to spread after the mitotic event (Figure 11H), meaning that it needed 4 h and 50 min to finalize a significantly, extremely slow division. This observation supports the conclusion that compounds in both S_5_ and S_2T_ fractions exerted a major antiproliferative effect, recommending them as potential anticancer therapeutic agents. There are very strong evidences on the fact that in those fractions was not identified other molecules, except amino acids and tionins. However, further investigations are needed to assert if the identified biological activity is due exclusively to amino acids, thionins or to other small molecules, as yet not identified by the analytical techniques used.

## 3. Experimental Section

### 3.1. General Information

All used reagents were analytical grade. Amino acids were acquired from Applichem (St. Louis, MO, USA) and Alfa Aesar (Ward Hill, MA, USA) and the solvents (methanol, acetone, dichloromethane, carbon tetrachloride, hexane, and *n*-butanol, petroleum ether, ethanol) from VWR (Wien, Austria). Pyridine and phenylisothiocyanate (PITC) were purchased from Alfa Aesar.

Plant roots and rhizomes were collected in February 2011, in Arieseni, Alba (Romania) and identified by Dr. Dana Bobit (vice-president of the Romanian Ethnopharmacology Society, Dacia Plant SRL Brasov, Romania). Voucher samples: 00321 and 00322 were deposited at the Botanical Garden Cluj Napoca, Romania.

### 3.2. Chromatographic Techniques

The content of amino acids and peptides from the hellebore samples was analyzed by high performance liquid chromatography (HPLC 3000, Ultimate, Dreieich, Germany) using a photodiode array detector and an EZ: faast 4u AAA-MS Column (250 × 3 mm ID). Qualitative analysis of free amino acids and peptides was performed on a GC-MS 7890A-5975C system (Agilent, Waldbronn, Germany) using the EZ: faast GC-MS free amino acids kit and ZB-AAA GC column (Phenomenex, Torrance, CA, USA). The employed analysis conditions were the standard conditions provided with the kit.

### 3.3. Spectroscopy Techniques 

Mass spectrometry was conducted on a High Capacity Ion Trap Ultra mass spectrometer (HCT Ultra, PTM discovery) from Bruker Daltonics (Bremen, Germany). All mass spectra were acquired in the mass range *m/z* 100–3000, with a scan speed of 2.1 scan per second.

### 3.4. Isolation Strategy for Amino Acids and Peptides 

The nature of the target bioactive compounds is influenced by the type of solvent employed. In general, a mixture of solvents was preferred to achieve the selective extraction of interested bioactive compounds from natural products. A preliminary sample was prepared using several solvents with different polarities (ethanol, dichloromethane, carbon tetrachloride, hexane, and *n*-butanol) in order to extract the corresponding phytochemicals from the hellebore extract. It was observed that in fractions with low and medium polarity (dichloromethane, carbon tetrachloride and hexane) lipophilic compounds were usually found, including: fatty acids, terpenes, steroids, peptides, depsipeptides, *etc.* High polarity fractions contained saponins, amino acids, alkaloids, sugars, *etc.* [24,25,26,33].

The solvent from a 50 mL hellebore hydroalcoholic (1.5:1 *v*/*v*; plant extract:alcohol ratio = 1:10) extract was removed under vacuum, the residue was dissolved in water and subsequently extracted with the following solvents: *n*-hexane (S_2_), carbon tetrachloride (S_3_), dichloromethane (S_4_) and *n*-butanol (S_5_). Each of the six organic fractions: S_1_ (ethanolic), S_2_ (hexane), S_3_ (CCl_4_), S_4_ (CH_2_Cl_2_), S_5_ (*n*-butanol), and S_6_ (aqueous) was analyzed by spectroscopic and chromatographic methods.

### 3.5. Derivatization of Standard Amino Acids

A pre-column derivatization method using PITC (phenylisothiocyanate) or Edman’s Reagent was carried out. The obtained phenylisothiocyanate derivatives (PITC-amino acids) were analyzed by reverse-phase high-performance liquid-chromatography [34,35,36,37,38,39]. This method was chosen because the derivatization reagent reacts easily with all amino acids in an alkaline milieu and produces stable products. A sample (1.5 μmol) of standard amino acids is mixed with 1 mL derivatization reagent: pyridine: H_2_O (40:60) and 15 mg PITC. The obtained mixture is heated at 40 °C for 1 h. Then, 1 mL H_2_O is added. The excess of PITC is removed by washing four times with 2 mL of benzene. The aqueous phase is evaporated and dried in vacuum. The residues are dissolved in 1.5 mL methanol and analyzed on HPLC.

### 3.6. Thionins

For the separation of thionins, the solvent from a 50 mL hellebore hydroalcoholic extract was removed under vacuum and the resulting residue was extracted successively with petroleum ether (20 mL) and acetone (20 mL). Both isolated fractions—S**_1T_** (petroleum ether) and S**_2T_** (acetone)—were investigated by analytical methods to reveal the presence of the isolated compounds.

### 3.7. GC-MS Separation Conditions 

The standard analysis conditions were according to instructions from the kit: Oven: 30 °C (hold 1 min) to 40 °C at 30 °C/min (hold 10 min) to 360 °C (hold 1 min); Equilibration time: 1 min; Injection: split 1: 15; 250 °C; 2 µL; Carrier Gas: Helium 1.1 mL/min; 110 °C; Inlet pressure: 5.824 kPa/min; Detector: MS; Mode: Scan Transfer Line Temperature: 250 °C; Analyzer Type: Electron Energy: 70 eV.

### 3.8. HPLC-DAD Separation Conditions 

The separation was performed by isocratic elution at Flow Rate: 0.6 mL/min., Col. Temp.: 35 °C and with UV detection (λ = 230 nm). Eluent A: H_2_O–acetonitrile–TFA (94.96% H_2_O, 5% acetonitrile, 0.037% TFA), Eluent B: H_2_O–acetonitrile–TFA (94.96% acetonitrile, 5% H_2_O, 0.037% TFA).

### 3.9. Mass Spectrometry Analysis 

Tandem mass spectrometry was carried out by collision-induced dissociation (CID) using He as the collision gas. For MS/MS sequencing, the precursor ions were selected within an isolation width of 2 u. Fully automated chip-nano ESI performed in a NanoMate 400 robot incorporating ESI chip technology (Advion BioSciences, Ithaca, NY, USA) coupled on a High Capacity Ion Trap Ultra mass spectrometer (HCT Ultra, PTM discovery, Bruker Daltonics). The robot was controlled and manipulated by the ChipSoft software (Advion BioSciences) operating under Windows. The position of the electrospray chip was adjusted to the sampling cone potential so as to give rise to an optimal transfer of the ionic species into the mass spectrometer. In order to avoid any contamination in all experiments, a glass-coated microtiter plate was used. Five μL aliquots of the working sample solutions were loaded onto the 96-well plate. The robot was programmed to take up the whole volume of sample, followed by 2 μL of air into the pipette tip and then deliver the sample on the inlet side of the microchip. Each nozzle has an internal diameter of 2.5 μm and under the given condition delivered a flow rate of about 200 nL/min. NanoESI process was initiated by applying voltages within 1.5 to 1.8 kV and a head pressure of 0.5–0.7 p.s.i. After spray initialization, infusion parameters were optimized: ESI voltage in pipette tip, voltage and desolvation gas flow. The values of Nano-ESI source parameter, ESI capillary, cone potential and desolvation gas (Nitrogen) were optimized to achieve an efficient ionization and produce the optimum transfer of ions in MS. Measurement parameters were: Capillary voltage 1 kV; Counter electrode voltage (cone voltage) 60 V; Acquisition time 2 min; Scan speed 2.1 scan/s; *m*/*z* 100–3000 mass range. The NanoMate HCT MS system was tuned to operate in the positive ion mode. This technique was chosen, as protein and peptide ionization shows high ionization efficiency in the positive ion mode. The source block, maintained at the constant temperature of 80 °C, provided an optimal desolvation of the generated droplets without a need of a desolvation gas. In all experiments, the desolvation gas was maintained at 30–50 liters per hour. For prevention of any cross contamination or carry-over, the pipette tip was ejected and replaced with a new one, after every sample infusion and MS analysis. All mass spectra were processed by the Data Analysis 3.4 software from Bruker Daltonik (Bremen Germany). Mass spectra were calibrated using sodium iodide as a calibrating agent. The accuracy on the determination of the average mass was 20 ppm, with a resolution of about 4000. Samples were dissolved in methanol at a concentration of about 5 pmol/μL. For an acquisition time of 2 min, the required volume of sample was about 2 pmols, a value which reflects a very high sensitivity analysis.

### 3.10. Study of the Biological Activity

HeLa cells were grown in DMEM-Ham F12 medium (Sigma-Aldrich, Munich, Germany), supplemented with 10% fetal bovine serum (Gibco, Basel, Switzerland) and 1% antibiotic-antimycotic solution (Lonza, Cologne, Germany). They were seeded at a density of 10^4^ cells/well in Hi–Q^4^ 35 mm dishes (Ibidi GmbH, Martinsried, Germany) After cell attachment (2 h), the medium was replaced with a fresh volume supplemented with various concentrations of S_5_ and S_2T_ lyophilized fractions (1, 2 and 4 μg/mL respectively, dissolved in culture medium). For the control group, the medium was aspirated and replaced with fresh standard medium. The dishes were then placed inside the Biostation IM equipment (Nikon Corp., Kawasaki, Japan), a mini-incubator with an incorporated optical system and a CCD camera for timelapse imaging. The cells were monitored for 24 h and images were collected every 10 min. To assess cell viability and proliferation, viable cells and mitoses were counted. For each experimental condition, images were collected from 5 different microscopic fields and the experiments were repeated three times. The wells which contained only the cells, without the added compounds, were considered as control.

## 4. Conclusions

This study was designed to investigate the chemical composition of extracts of a valuable medicinal plant, the hellebore, with therapeutic effects known since long ago in traditional medicine. The amino acids and peptides contained in a hellebore hydroalcoholic extract were identified and isolated. An appropriate isolation strategy was developed that can be applied for the isolation of such compounds from complex mixtures. The presented results indicate that the new isolation strategy and the feasibility of the developed characterization method show great potential as efficient separation methods for amino acids and peptides from natural products. The methods used in our study to isolate various fractions of the hellebore extract did not affect the antiproliferative activity, as shown by treating HeLa cells and monitoring their behavior by time lapse videomicroscopy.
