# Expression of Concern: Segneanu *et al*. *Helleborus purpurascens*—Amino Acid and Peptide Analysis Linked to the Chemical and Antiproliferative Properties of the Extracted Compounds. *Molecules* 2015, *20*, 22170–22187

**DOI:** 10.3390/molecules21060725

**Published:** 2016-06-02

**Authors:** Derek J. McPhee, Isabel C. F. R. Ferreira

**Affiliations:** 1 MDPI AG. Molecules Editorial Office, Klybeckstrasse 64, 4057 Basel, Switzerland; 2 Mountain Research Centre, School of Agriculture, Polytechnic Institute of Bragança, Campus de Santa Apolónia, 1172, 5300-253 Bragança, Portugal

As Editor in Chief of Molecules and Academic Editor of the article [1], respectively, we wish to advise readers that Molecules has been informed that its authorship is currently in dispute and the subject of a formal lawsuit. Following the recommendations of the Committee on Publication Ethics (COPE), of which MDPI is a member, until all outstanding legal issues are resolved, we cannot comment nor take any further action, and the purpose of this Expression of Concern is simply to alert readers of these facts so that they may act accordingly.

Addendum (10 January 2018): An Editorial has been published to update this Expression of Concern. No changes have been made to the original published paper. See http://www.mdpi.com/1420-3049/23/1/136.
